# Percutaneous Atrial Septal Defect Closure Using the Occlutech Figulla Device in Adults: More than 800 Patient-Years of Follow-Up

**DOI:** 10.1155/2020/7136802

**Published:** 2020-02-14

**Authors:** R. J. R. Snijder, L. E. Renes, D. Bosshardt, M. J. Suttorp, J. M. ten Berg, M. C. Post

**Affiliations:** ^1^Department of Cardiology, St. Antonius Hospital, Nieuwegein, Netherlands; ^2^Department of Emergency Medicine, St. Antonius Hospital, Nieuwegein, Netherlands

## Abstract

**Purpose:**

The Occlutech Figulla occluder has been proven safe and effective at midterm follow-up after percutaneous atrial septal defect (ASD) closure. We describe the safety and efficacy at long-term follow-up in adults.

**Methods:**

All consecutive adult patients that underwent ASD closure between 2008 and 2015 were included. All complications were registered. Residual left-to-right shunt (LRS) was diagnosed using color-Doppler transthoracic echocardiography (TTE). Right-to-left shunting was diagnosed using contrast TTE. Successful closure was defined as no LRS at follow-up.

**Results:**

In total, 166 patients (mean age 56.7 ± 16.1 years; 62% female) underwent percutaneous ASD closure using the Occlutech Flex I (70%) or Flex II (30%) device (diameter 24 mm; range 10–40 mm) under general anaesthesia and transoesophageal echocardiographic guidance. Long-term follow-up data were available for 144 patients (87%) with a mean follow-up of 5.9 ± 2.6 years, a total of 814 patient-years. During hospitalization, device embolization occurred in three patients (1.8%) with successful extraction in all. During the long-term follow-up, 15 patients (9.8%) suffered new-onset atrial fibrillation and stroke occurred in 2.1%. There was no residual LRS at 12-month follow-up. No device embolization occurred during the long-term follow-up.

**Conclusion:**

Percutaneous ASD closure using the Occlutech device appears to be safe at long-term follow-up with a high successful closure rate at one year.

## 1. Introduction

Atrial septal defects (ASDs) are a common cardiac congenital finding with complications due to the left-to-right shunt (LRS). Percutaneous closure of an ASD is considered to be the first choice and has been proven safe and effective using different devices [1, 2]. Worldwide, there is a lot of good experience with the Amplatzer septal occluder. The Occlutech Figulla septal ASD occluder (OFSO) was developed to improve the feasibility of implantation and lower the rate of possible complications such as device thrombosis with an absent left atrial hub. In the past decade, three generations of the OFSO device were developed. All generations have proven to be safe and effective with a low complication rate during short- and midterm follow-up [3–6]. However, little is known about the long-term complications of this extensively used device. We describe the safety and efficacy of percutaneous ASD closure in adults using the OFSO after more than 5 years of follow-up.

## 2. Methods

### 2.1. Population

All consecutive adult patients, ≥18 years of age, that underwent percutaneous ASD closure with the Occlutech Figulla ASD Flex I or Flex II device (Occlutech®) in St. Antonius Hospital, Nieuwegein, Netherlands, between 2008 and 2015 were included. The local ethics committee approved the study (registration number R&D/Z16.054).

### 2.2. Closing Procedure

Percutaneous closure was performed under general anaesthesia using transesophageal echocardiography (TEE) and balloon sizing as reported earlier [6]. Depending on the size and morphology of the ASD, device size was chosen by the interventional cardiologist. The initial success rate was defined as successful implantation.

### 2.3. Follow-Up

All complications were registered. All patients were discharged on antiplatelet therapy: clopidogrel (75 mg) for 4 weeks and aspirin (80–100 mg) for at least 6 months. If a patient was under anticoagulation therapy, only clopidogrel was associated for 4 weeks. Routine follow-up after closure was scheduled at 1, 6, 12, and 24 months using color-Doppler echocardiography to determine the presence of a residual LRS. Bubble contrast, at rest and after Valsalva, was used to identify a right-to-left shunt (RLS). The size of the RLS was classified by the number of bubbles in the left ventricle on a still frame and graded as minimal (<30 bubbles), moderate (30–100 bubbles), and large (>100 bubbles) [7]. Successful closure was defined as no residual LRS shunt using color-Doppler. Firstly, the electronic patient file was studied to find out whether the patient had a recent follow-up (within 6 months). If not, the patient was asked to participate in an interview by phone. A questionnaire was created together with the interventional and congenital cardiologists with standard follow-up questions. Besides the complaints that were mentioned by the patient, questions were asked about cerebral ischemic events (TIAs or stroke), presence of supraventricular tachycardias, and hospital admissions. If a patient was admitted to another hospital, that hospital was contacted to retrieve the necessary information. When it was unable to get in contact with the patient, the general practitioner was contacted. The questions were standardized to create complete and uniform data.

### 2.4. Statistical Analysis

Descriptive statistics were used for patients' characteristics. Continuous variables with normal distribution are presented as mean ± standard deviation. All statistical analyses were performed using the SPSS software (version 24.0 for Windows).

## 3. Results

### 3.1. Patient Characteristics

In total, 166 adult patients (62% women; mean age 56.7 ± 16.1 years) underwent percutaneous ASD closure using the Occlutech Figulla device between 2008 and 2015. During that study period, no other closure device had been used. The Occlutech Figulla Flex I was implanted in 116 patients (69.9%) and the Occlutech Figulla Flex II in 50 patients (30.1%). There were no differences in baseline characteristics between patients who received the different devices. Patient characteristics of the whole group are summarized in Table 1.

### 3.2. In-Hospital Complications

Device implantation (mean diameter 24 mm; range 10–40 mm) was initially successful in all patients. However, device embolization occurred in three patients (1.8%) within 24 hours after implantation. After a failed attempt to retrieve the devices percutaneously, they were extracted surgically followed by closure of the ASD. Balloon sizing (using the Amplatzer sizing balloon II, 34 mm (Abbott)) was performed in almost all of the patients. However, another sizing balloon (NuMED PTS-X 40 mm/5 cm-sizing balloon (NuMED Inc.)) had been used, in 2 out of the 3 device embolizations, leading to an inappropriate sizing due to inadequate calibration. In the other patient, a deficient aortic rim was present.

One patient (0.6%) suffered a significant amount of pericardial effusion (PE) a few hours after closure and needed percutaneous drainage. The next day, a second TTE showed no PE. The drain was retrieved, and the TTE was repeated a couple of hours thereafter. Again the TTE showed no PE. The patient was discharged, and after one and four weeks, no recurrent PE was found by TTE.

### 3.3. Follow-Up

At 6-month follow-up, device thrombosis occurred in one patient (0.6%) who was noncompliant for oral anticoagulation therapy prescribed for recurrent idiopathic deep venous thrombosis. The device was extracted surgically, followed by closure of the ASD. All patients in whom a complication occurred recovered well. There were no significant differences in complications between both devices. In-hospital complications are shown in Table 2.

In total, new-onset AF occurred in 9.8% of the patients during the overall follow-up. Recurrent thrombo-embolic events occurred in seven patients (3 stroke, 2.1%; 4 TIA, 2.8%) during the total follow-up of almost 6 years. Details of these patients are summarized in Table 3.

Six patients died during follow-up. Three patients died in 15 months, two in 18 months, and one in 33 months after closure, respectively. One patient died due to the complications of liver cirrhosis. This patient had no evidence of pulmonary hypertension on TTE prior to closure. Another patient died because of systemic inflammatory response syndrome due to orbital cellulitis. He had a slightly elevated right ventricular systolic pressure (RVSP 40 mmHg) prior to closure. The third patient died after a tuberculosis infection. Before closure, this patient had an elevated RVSP (46 mmHg), which decreased after closure to 34 mmHg. A 70-year-old male, with a history of arterial hypertension and coronary artery disease, died due to sever systolic heart failure after myocardial infarction. There was no thrombus on the device and no elevated RVSP or secondary signs of pulmonary hypertension prior to closure. The cause of dead of the other two patients is unknown. No device-related cause was suspected after interviewing the families and general practitioners. One of these patients had a RVSP of 43 mmHg without secondary signs of pulmonary hypertension, and the other patient had normal pressures and no signs of pulmonary hypertension either. However, no autopsy was performed. Therefore, a device-related cause could not be excluded.

TTE was performed in 71 patients (49.3%) at 12-month follow-up. Follow-up information was available for 144 patients at long-term follow-up (mean 5.9 ± 2.6 years); data (interview or TTE) could not be retrieved for 22 patients. There was no recurrent LRS at the latest follow-up. There were no significant differences between both devices at long-term follow-up. Long-term follow-up data are presented in Table 4.

## 4. Discussion

Percutaneous ASD closure using the Occlutech device is safe and effective during a long-term follow-up of more than 800 patient-years.

### 4.1. Complications

In the current literature, an overall initial successful device implantation using the Occlutech device had been described between 94% and 99% of the procedures [2–4, 8, 9]. Complications that are described are related to the invasive procedure itself, such as groin hematoma and pericardial effusion. Other complications are related to the specific procedure using a device. Firstly, a device embolization is described between 0% and 2.6% for which percutaneous or surgical retrieval was necessary [2–4, 6, 9–13]. Haas et al. described that 1291 patients underwent successful percutaneous ASD closure with the Occlutech device. A device embolization occurred in 20 patients (1.6%) during hospitalization. There were another five embolizations during a mean follow-up of 2.7 years. Predictors for embolization are the absence of balloon sizing and the use of larger devices [9]. Kim et al. studied both the Occlutech and Amplatzer devices in ASD closure and found an embolization in one patient (1.0%) using the Amplatzer device and none in the Occlutech group [14]. More recently, Kenny et al. described a randomized controlled multicenter trial comparing the Occlutech and Amplatzer devices in 176 patients (both children and adults). They found a more successful device placement (99% vs. 90%) and early efficacy (94% vs. 90%) and less in-hospital major complications (5.6% versus 9.8%) using the Occlutech versus the Amplatzer device [12]. Secondly, an atrioventricular block occurs between 0% and 3.4%, mostly caused by oversizing the device [2, 3, 6, 8, 9]. This complication was often resolved after extraction of the device. An atrioventricular block occurred in seven patients (0.5%) during hospitalization in the study by Haas et al., of which five needed a smaller device. Thirdly and less common is device thrombosis; this occurred between 0% and 1% [3–5, 7, 8]. There was no device thrombosis in the study by Haas et al. Fourthly, pericardial effusion after percutaneous ASD is found in about 1.9%. The etiology of the pericardial effusion is unclear, but predictors are older age and higher body surface area at closure [13].

In our study, implantation was successful in 98%. Embolization occurred in 1.8%, which is similar when compared to the other studies. All three devices were extracted surgically, and the ASD had been closed without complications. There was one patient, noncompliant for Coumadin, who suffered a device thrombosis (0.6%). One patient suffered pericardial effusion without evidence of device perforation. As mentioned above, there were no significant differences in complications between the second- and third-generation devices. As mentioned earlier, Haas et al. described a large patient group of both children and adults. The median age was 26 years (range 0.3 to 83 years), which is significantly lower than our population. Furthermore, the first-generation Occlutech septal occluder (OSO) was used in 42% of patients. In our study, only second- and third-generation devices were used. Though, our results are similar to those described by Haas et al., cautiousness is necessary when comparing both studies.

### 4.2. Cerebrovascular Events

In the literature, cerebrovascular events after closure are rare and occur between 0% and 2.3% [3–5, 8–11]. None of the patients described in the four studies with a total number of 501 patients suffered a TIA or stroke after closure [3–5, 8]. The study by Haas et al. described four patients (0.3%) who suffered a TIA or minor stroke [9]. Takaya et al. and Wang et al. described a stroke rate after closure between 1.2% and 2.2%, which is higher when compared to other studies. Though it is unclear what causes this higher cerebrovascular event rate, the age of the patients in both studies is significantly higher when compared to the other studies and might be the reason for this difference [10, 11]. In our study, a stroke rate of 2.1% was found during the long-term follow-up without a device-related cause. One patient had a minimal RLS at follow-up; however, the clinical relevance of this small RLS is unclear. Patient characteristics such as age and the presence of cardiovascular risk factors together with the longer follow-up duration could be the explanation. As described above, the age and cerebrovascular rate in the study by Takaya et al. and Wang et al. were high as well. A higher age is a predictor for cerebrovascular events after closure and in the overall population as well [15]. The cerebrovascular event rates were similar between both devices.

### 4.3. Arrhythmias

New-onset AF or atrial flutter is a known complication after percutaneous ASD closure and varies in studies between 0% and 4.7%. Supraventricular tachycardias (SVT) occurred in 4.7% in the study by Haas et al. [9]. There was no new onset of SVTs reported during the follow-up in the study by Pedra et al. and Roymanee et al. In 1% of the patients from the study of Aytemir et al., an SVT was diagnosed [4, 5, 8].

In our study, new-onset AF occurred in 5.4% during the first year and in 4.4% at long-term follow-up, which is slightly higher when compared to the literature. A possible explanation is the higher age of our patients when compared to the other studies. There were new-onset SVTs in 11.7% in the study by Wang et al., which is much higher than the other studies [11]. This study also included patients with a higher age. Literature showed a higher incidence of AF in older patients and this might be the explanation [16]. There were no significant differences between both devices which were used. In most studies, new-onset SVTs were diagnosed according to symptoms or coincidental findings on ECG during regular outpatient visits. Therefore, it is difficult to know the exact incidence of new-onset SVTs after closure.

### 4.4. Residual Shunting

Successful closure using the Occlutech device varies between 90% and 100% at different follow-up times [2, 3, 8, 9]. In our study, successful closure was achieved in all patients that underwent TTE at 12-month follow-up. Though TTE was only performed in 49% of patients, the success rate might be overestimated. Secondly, the LRS rate could be higher if TEE had been used instead of TTE. However, a small residual LRS found by TEE would probably have no clinical importance.

## 5. Limitations

Our study was an observational, single-center study describing the Occlutech device without comparison to other devices. Further, TTE was used at follow-up, making it possible to underestimate the residual LRS rate. However, it is unclear whether such a small LRS would be of clinical importance. There are no data about residual LRS at long-term follow-up, but TTE at discharge or one-year follow-up showed no LRS, making it unlikely that it would be found at long-term follow-up. We used telephone interviews for obtaining long-term data. This might be insufficient, and data about TTE are therefore not available.

## 6. Conclusion

Percutaneous ASD closure using the Occlutech device has a high successful closure rate at 12 months and appears to be safe at long-term follow-up with a low complication rate. However, more follow-up data are needed to make a reliable conclusion.

## Figures and Tables

**Table 1:** Baseline characteristics.

Number of patients	166
Age (years)	56.7 ± 16.1
Female, *n* (%)	103 (62.0)
BMI (kg/m^2^)	25.6 ± 4.5
Systolic blood pressure (mmHg)	129.3 ± 17.0
Diastolic blood pressure (mmHg)	79.2 ± 9.3

Risk factors and comorbidities, *n* (%)	
Smoking	23 (13.9)
Diabetes	11 (6.6)
Arterial hypertension	50 (30.1)
Hypercholesterolemia	39 (23.5)
CAD	10 (6.0)
History of AF or AFl	19 (11.4)

Indication for closure, *n* (%)	
RV volume overload	104 (62.7%)
Cryptogenic stroke/TIA	47 (28.3%)
Others	15 (9.0%)

Echocardiography	
RVSP (mmHg)	25.4 ± 7.8
Peak TRV (m/sec)	2.5 ± 0.4
Peak TRV (2.9–3.4 m/sec), *n* (%)	24 (14.5)
Peak TRV (>3.4 m/sec), *n* (%)	4 (2.4)

Mean follow-up (years)	5.9 ± 2.6

Data are presented as mean ± SD or number (percentage). BMI, body mass index; CAD, coronary artery disease; AF, atrial fibrillation; AFl, atrial flutter; RV, right ventricle; TIA, transient ischemic attack; RVSP, right ventricular systolic pressure.

**Table 2:** Periprocedural characteristics.

General anaesthesia, *n* (%)	166 (100)
TEE guiding, *n* (%)	166 (100)
ASD diameter on TEE (mm)^+^	15.6 ± 6.1
ASD balloon sizing (mm)^+^	20.9 ± 6.7
Device	
Occlutech Flex I, *n* (%)	116 (69.9)
Occlutech Flex II, *n* (%)	50 (30.1)
Device diameter (mm)^*∗*^	24 (10–40)
In-hospital complication	
Device embolization	3 (1.8%)
Pericardial effusion	1 (0.6%)
New-onset AF	3 (1.8%)
Groin hematoma	13 (7.8%)
TTE shunt, *n* (%)	
Color-Doppler	23 (14.1%)

^*∗*^Data are presented as median (range). ^+^Data are presented as mean± standard deviation. TEE, transesophageal echocardiogram; ASD, atrial septal defect; AF, atrial fibrillation; TTE, transthoracic echocardiogram.

**Table 3:** Thrombo-embolic events during follow-up.

Patient, n	Age, years	Sex	Stroke/TIA	History of stroke/TIA	Time after closure	AF history	RLS	Device thrombus
1	46	Male	Stroke	Stroke	<12 months	No	Minimal	No
2	74	Female	Stroke	No	<12 months	No	No	No
3	26	Female	TIA	Stroke	<12 months	No	Moderate	No
4	71	Female	Stroke	TIA	>12 months	No	No	No
5	79	Female	TIA	No	>12 months	No	No	No
6	46	Female	TIA	TIA	>12 months	No	No	No
7	58	Female	TIA	TIA	>12 months	No	No	No

TIA, transient ischemic attack; AF, atrial fibrillation; RLS, right-to-left shunt.

**Table 4:** Efficacy and safety during follow-up.

	<12-month follow-up	≥12-month follow-up
Number (*n*)	166	144
Complications, *n* (%)		
TIA	1 (0.6%)	3 (2.1%)
Stroke	2 (1.2%)	1 (0.7%)
AF	9 (5.4%)	6 (4.4%)
Death	0 (0%)	6 (4.4%)
TTE available (*n*)	163 (98.2%)	71 (49.3%)
LRS, *n* (%)	0 (0%)	0 (0%)
RLS, *n* (%)		
No shunt	113 (69.3%)	49 (69.0%)
Minimal	30 (18.5%)	16 (22.5%)
Moderate	15 (9.2%)	5 (7.1%)
Severe	5 (3.0%)	1 (1.4%)

TIA, transient ischemic attack; AF, atrial fibrillation; TTE, transthoracic echocardiography; RLS, right-to-left shunt.

## Data Availability

The data used to support the findings of this study are available from the corresponding author upon request after the author gets approval of the ethics committee.
